# Mechanistic insights into the key role of methylammonium iodide in the stability of perovskite materials†

**DOI:** 10.1039/d3ra01304a

**Published:** 2023-07-10

**Authors:** Negin Sabahi, Hashem Shahroosvand

**Affiliations:** a Group for Molecular Engineering of Advanced Functional Materials, Department of Chemistry, University of Zanjan Iran shahroos@znu.ac.ir

## Abstract

The possible mechanisms damaging perovskite solar cells have attracted considerable attention in the photovoltaic community. This study answers specifically open problems regarding the critical role of methylammonium iodide (MAI) in investigations as well as stabilizing the perovskite cells. Surprisingly, we found that when the molar ratio between PbI_2_ : MAI precursor solution increased from 1 : 5 to 1 : 25, the stability of perovskite cells dramatically increased over time. The stability of perovskite in the air without any masking in the average stoichiometry was about five days, while when the amount of MAI precursor solution increased to 5, the perovskite film was unchanged for about 13 days; eventually, when the value of MAI precursor solution enhanced to 25, the perovskite film stayed intact for 20 days. The outstanding XRD results indicated that the intensity of perovskite's Miler indices increased significantly after 24 h, and the MAI's Miler indices decreased, which means that the amount of MAI was consumed to renew the perovskite crystal structure. In particular, the results suggested that the charging of MAI using the excess molar ratio of MAI reconstructs the perovskite material and stabilizes the crystal structure over time. Therefore, it is crucial that the main preparation procedure of perovskite material is optimized to 1 unit of Pb and 25 units of MAI in a two-step procedure in the literature.

## Abstract 2

The possible mechanisms damaging perovskite solar cells have attracted considerable attention in the photovoltaic community.

## Graphical abstract

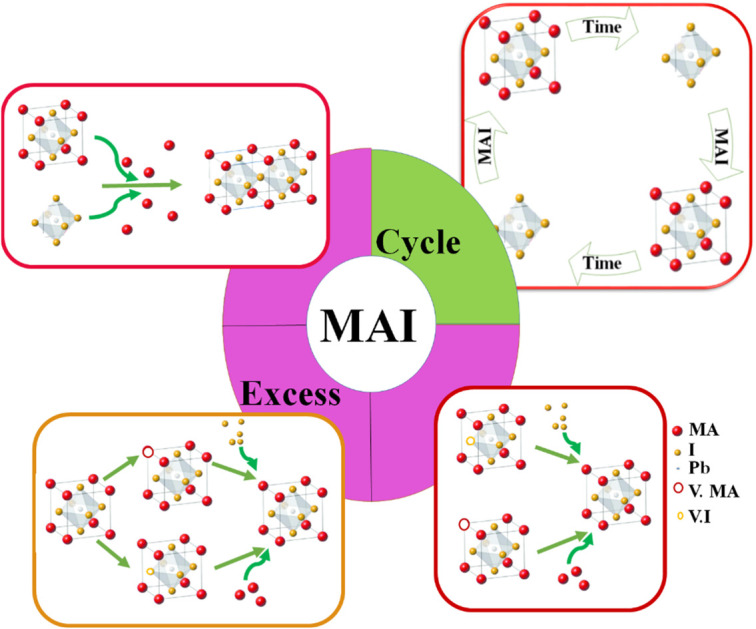

## 1. Introduction

Substances with the chemical formula ABX_3_ and cubic structure are known as perovskite materials,^1^ in which A and B can be inorganic cations with different ion capacities and radii.^2^ In general, interesting physical properties such as magnetic resistance,^3^ ferroelectricity,^1^ and superconductivity^2^ have been extensively discovered in perovskites. On the other hand, perovskite has a crystal structure that is sensitive to light, which has led to a breakthrough in solar energy generation. In this context, perovskite solar cells (PSCs) have received significant attention due to their impressive energy conversion efficiency (PCE) of up to 25.8% and their easy and low-cost preparation.^4,5^ In addition to existing advances, the presence of lead in the center of the perovskite cell as a toxic metal, the stability of the cell over time, and the efficiency of the structure are among the things that need improvement.^6^ The most important things attempted are the following; 1- replacement with other cations such as triple cation alternatives,^7^ 2- increasing the particle size and reducing grain boundaries^8,9^ using different additives, 3-ease of movement of electric charge carriers,^10–12^ 4- reducing the effect of hysteresis (current reduction)^13^ and charge recombination^14^ by controlling crystal growth.

In this area, simple, cost-effective, and straightforward ways to make high-quality perovskite films are by incorporating additives that remarkably improve the photovoltaic performance through the following strategies.^15^ First, the modulation of perovskite film morphology through the optimization of colloid size and mediation for nucleation as well phase stabilization.^16^ Second, adjustment of energy levels from 1.2 to over 3 eV for charge extraction.^17^ Second, the reduction of non-radiative recombination and elimination of hysteresis (reduction of flow). Generally, common additives used for the above quarries include salts, organic and inorganic molecules, polymers, and nanoparticles.^18^ However, additives that have been studied so far are very extensive such as thiocyanates,^19^ excess lead iodide,^20^ methyl ammonium iodide,^21^ chloride,^22^ bromide,^23^ solvents,^24^ Lewis acids and bases,^25^ hydrophobic materials,^26^ oxygen and silica,^27^ two-dimensional perovskites,^28^ and quantum datasets.^29^ The variety of additives may be related to the ability to coordinate lead cation (Lewis acid) and iodide anion (Lewis base) in the perovskite structure.^30^ It is worth noting that a deep understanding of the relationship between the physicochemical properties of the additives and the improved quality of the perovskite film is essential for developing new additive systems,^7^ which are expected to be enhanced by surface engineering pathways. Few articles have investigated the effect of methyl ammonium iodide (MAI) in various conditions (Table S1†); for instance, N. Adhikari *et al.* reported the influence of increasing the amount of water in a solution of MAI in isopropyl alcohol (IPA), which increases the grain size.^31^ I. Levchuk *et al.* found that MAI impurities such as hypo phosphorus acid affect perovskite film quality.^32^ Additionally, Y. Cheng *et al.* confirmed that the loading time of MAI affects the electrical and conductive properties of perovskite thin films.^33^ Based on the observation by K. S. Kim *et al.*, adding excess MAI to CsPbI_2_Br film controls the crystal size of perovskite units.^34^ K. Liao *et al.* reported that the extra-stoichiometric film of MAI forms a large-grain perovskite membrane.^35^ Moreover, A. Kogo, *et al.* and F. Sahli *et al.* indicated that (MAI) post-treatment and using the vapor transport deposition technique of methylammonium iodide, respectively, can improve the PCE of PSCs.^36,37^ Finally, L. Qiu *et al.* reported that film growth can be controlled with MAI and a green antisolvent *sec*-butyl alcohol.^38^

In light of the studies mentioned above, there is no report on the influence of MAI on the stability of PSC. Therefore, this study will investigate the effect of changing the amount of MAI on the stability, purity, and crystallization of perovskite. In this regard, the following insights will be addressed; first, the presence of excess MAI gradually repairing perovskite crystal defects that may have occurred during or after the preparation of perovskite film due to the decomposition process. Second, the excess MAI continues the reaction and forms new perovskite grains from PbI_2_ molecules that did not react. Third, the number of layer depositions of MAI can increase the dark brown perovskite layer after the perovskite coating turns yellow.

## 2. Experimental

### 2.1. Materials and equipment

All materials are provided by Merck and Sigma Aldrich with a purity of 99%. Ultraviolet-visible (UV-vis) spectra were registered in an Ultrospec3100 pro spectrophotometer and UV-vis NIR, Cary5E, Varian, USA. X-ray diffraction analysis (XRD) was performed using an XRD Bruker D8 Model: advance made in Germany, powder X-ray diffractometer using Cu Kα radiation. The digital photography imaging was recorded by Samsung M51 mobile phone. Field emission scanning electron microscope (FE-SEM) images were obtained using FESEM MIRA3TESCAN-XM field-emission scanning electron microscope. Optical microscope images were obtained by a Microscope BeEL, MPL-15, Made in Italy. X-ray photoelectron spectra (XPS) were measured by XPS Bes Tec, vacuum: 10^−10^, anode: Mg, energy: Kα, 1253.6 eV made in Germany.

### 2.2. Synthesis method

In this project, the perovskite thin film samples were prepared by the deposition of PbI_2_ and then MAI with the ratio of 1, 5, and 25.

For the first MAI (1), 460 mg PbI_2_ was dissolved in 1 mL dimethylformamide (DMF) and spin-coated on glass in the glove box, then 6 mg MAI was dissolved in 1 mL of 2-propanol and spin-coated on prepared PbI_2_ and placed on a hotplate at 100 °C for 20 s. The color of the deposited layer went from yellow to dark brown, which indicated the formation of perovskite film.

For the second MAI (2), all steps of MAI (1) were repeated, except the amount of MAI was increased from 6 mg mL^−1^ to 30 mg mL^−1^.

The procedure of the third MAI (3) is the same as MAI (1), except that the amount of MAI was raised from 6 mg mL^−1^ to 150 mg mL^−1^. All steps of preparation of perovskite film have been done in a glove box without any encapsulation and masking. All samples were kept at room temperature in the air and mild light for testing their stabilities.

## 3. Results and discussion

### 3.1. XRD and XPS analyses

**Fig. 1:**
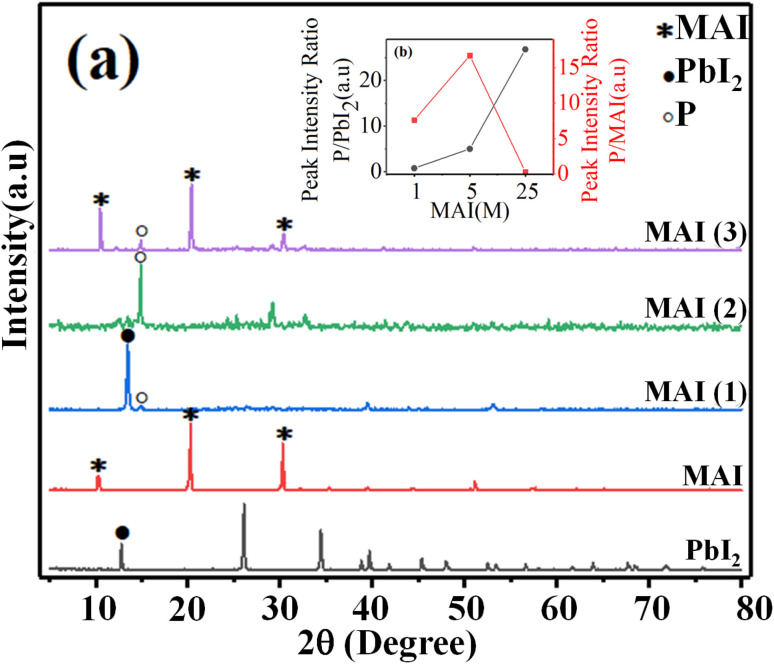
(a) XRD pattern of (PbI_2_), (MAI), and perovskite thin films with different ratios of MAI, MAI (1), MAI (2), MAI (3), (b) intensity diagram of XRD peaks of the perovskite to the PbI_2_ peak was located at 14.9 and 12.7°, P/PbI_2_ (blackline), intensity diagram of perovskite peak to MAI peak at 14.9 and 10.5°, P/MAI (redline).

First, the crystal structure and purity of the perovskite compound were identified by XRD analysis in order to confirm the excess precursor materials in it. The XRD pattern of all samples is shown in Fig. 1(a). The perovskite film CH_3_NH_3_PbI_3_ prepared by the spin-coating method (the details will be given in the Experimental section) indicates the presence of raw material (PbI_2_). The peak of surface of 001 corresponds to PbI_2_ located at 12.7°, which can be seen in MAI (1). When the MAI molar ratio is increased to 5 times the initial value, the ratio of the perovskite peak with Miller index 110, located at 14.9°, to the PbI2 peak with Miller index 001, located at 12.7°, 5 has been estimated, which means that the amount of PbI2 has decreased dramatically and the purity of perovskite has increased obtained.

When the molar ratio of MAI increased to twenty-five times the initial value, the MAI peak corresponding to the surface (001), (002), and (003) located at 10.5°, 20.4°, and 30.3°, respectively, dramatically increased.

Moreover, the intensity of perovskite peak to MAI increased when the concentration of MAI to PbI_2_ increased 5 times, while when the concentration of MAI increased 25 times, the ratio of perovskite to MAI peak decreased significantly. These surprising results indicated that the high-purity perovskite, which has the lowest MAI and PbI_2_ peaks, was obtained when the concentration ratio between MAI and PbI_2_ was five times the initial value (MAI/PbI_2_ = 5).

**Fig. 2:**
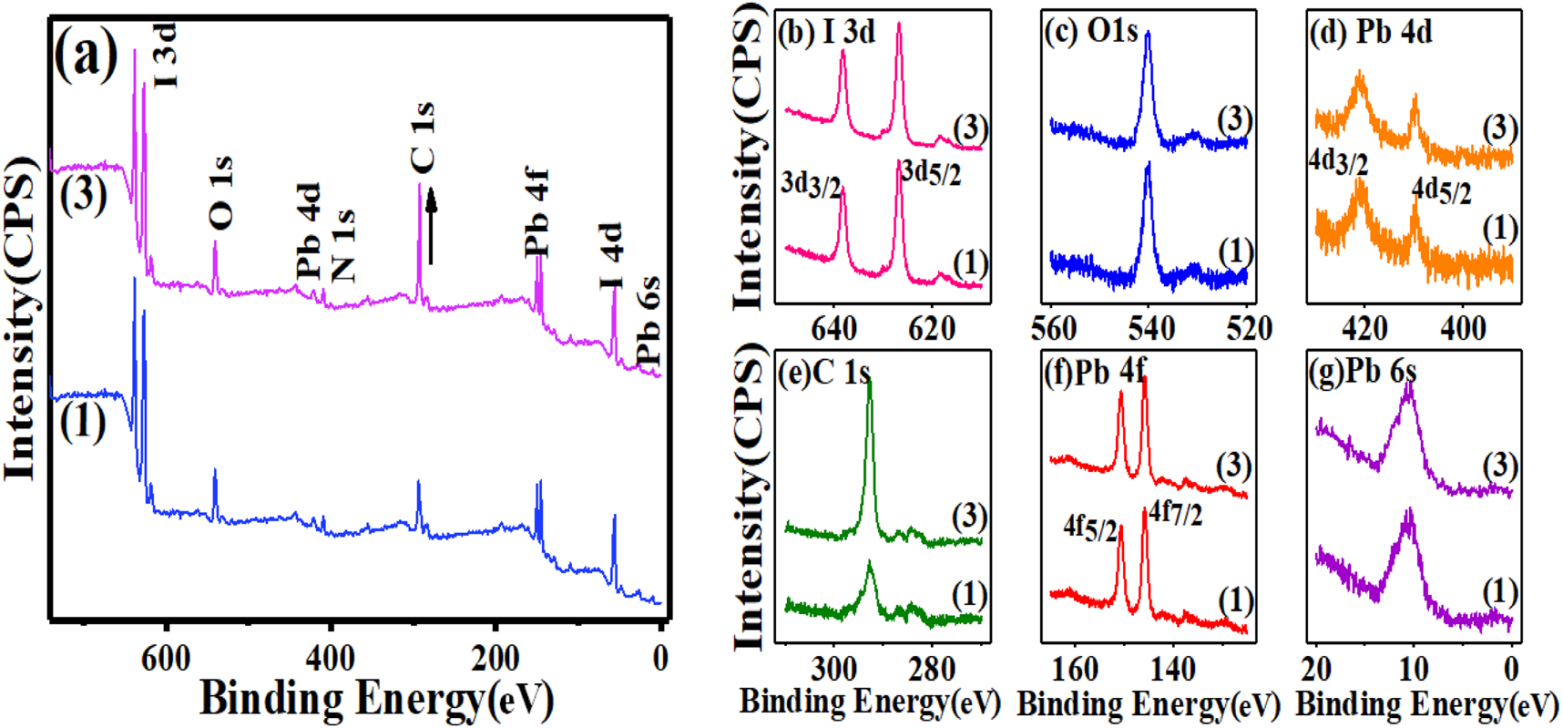
XPS survey spectra of the (a) perovskite films MAI (1) and MAI (3), (b) multiplicity 5/2 and 3/2 of 3d orbitals of iodine atoms, (c) 1s orbital of oxygen atoms, (d) multiplicity 5/2 and 3/2 of 4d orbitals of lead atoms, (e) 1s orbital of carbon atoms, (f) multiplicity 7/2 and 5/2 of 4f orbitals of lead atoms, (g) 6s orbital of lead atoms.

For a deeper insight into the above observation, X-ray photoelectron spectroscopy (XPS), a quantitative technique for measuring the elemental composition of the surface of a material, which also determines the binding states of the elements, was carried out. For instance, Y. Zhong *et al.* indicated the effect of phenyl-C61-butyric acid methyl ester (PCBM) as an additive on the perovskite film, by which PCBM molecules would diffuse into the perovskite film, which inactivates iodine-related defects to significantly reduce iodine ions/vacancies and reduce internal field modulation as well as surface barriers.^39^ A recent study of element distribution on the surface of different perovskite layers using XPS survey spectra by Q. Zhou *et al.* showed that 2-(4-fluorophenyl) ethyl ammonium iodide (FPEAI) forms a one-dimensional layer on a three-dimensional perovskite layer to protect the perovskite film.^40^ As shown in Fig. 2(a)–(g), the XPS spectra of the comparison of perovskites containing MAI (1) and MAI (3) indicated the isolated six peaks of lead atoms from 5 to 450 eV. One peak at 9.8 eV of 6s orbitals and two peaks at 145.9, 150 eV corresponding the 7/2 and 5/2 multiplicity of 4f orbitals, as well two peaks at 421.1 and 409.1 eV of the 5/2 and 3/2 multiplicity of 4d orbitals are clearly demonstrated, which approved the pattern of perovskite film. The presence of the 1s orbital of oxygen and carbon atoms as well as peaks at 539.9 eV and 292.8 eV were appropriately confirmed by the reference pattern, too. There was no change in the intensity and position of all peaks with variable concentrations of MAI compared to perovskite prepared with MAI (1), except in the excess MAI (3), where the orbital intensity of carbon 1s increased significantly, which can be attributed to many species of MAI. As observed in Fig. 2(a)–(g), there are no abnormalities in the perovskite crystal structure, while surprisingly, charging the MAI on the MAI (3) improves the stability of the perovskite over a long time, which will be discussed in the following sections.

### 3.2. FE-SEM analysis

**Fig. 3:**
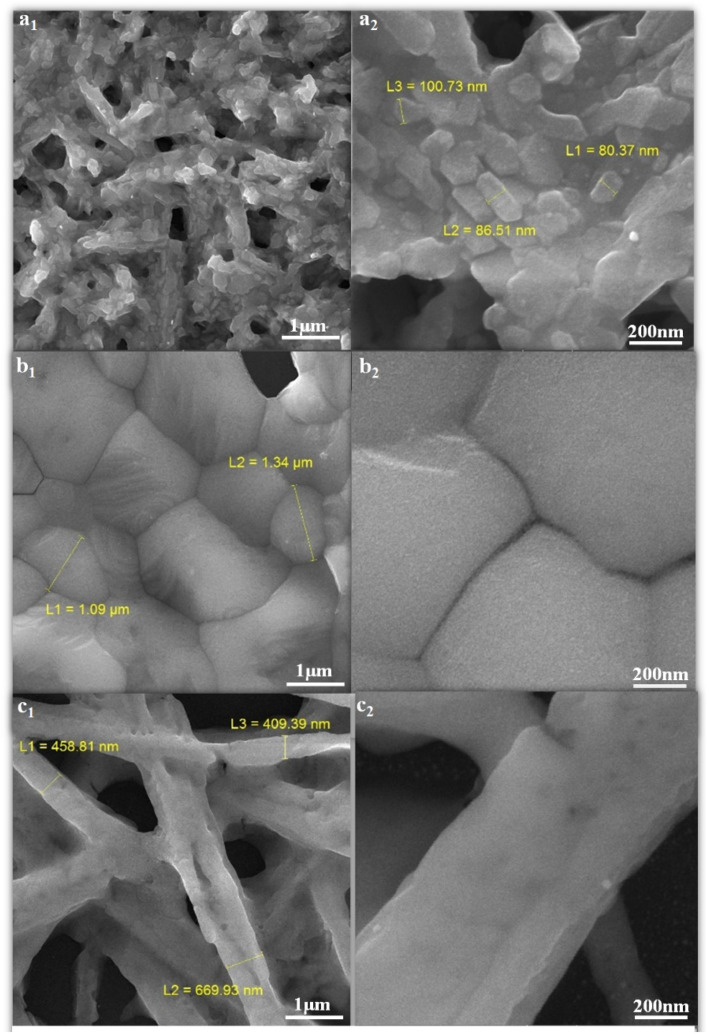
FE-SEM images of perovskite thin films with different ratios of (MAI), (a_1_ and a_2_) MAI (1), (b_1_ and b_2_) MAI (2), (c_1_ and c_2_) MAI (3).

FE-SEM images of perovskites prepared with different ratios of MAI, MAI (1) 3a, MAI (2) 3b, and MAI (3) 3c are shown in Fig. 3. An obvious change is observed with changing the concentration of MAI on perovskite morphology as follows: when the concentration of MAI was fixed at a concentration of 1 M, the perovskite grains were in the range of 100 nm, and by increasing the concentration to 5 M, the grain size reaches 1 μm, and finally, the concentration of MAI of 25 M appears as needles with a diameter of 400 nm. Confirming the effect of concentration on morphology, interesting research by Y. Rahaq *et al.* using SEM analysis has shown that low concentrations of MAI reduce the density of nucleation, and consequently, the density of perovskite formation will be decreased.^41^

### 3.3. The optical properties

**Fig. 4:**
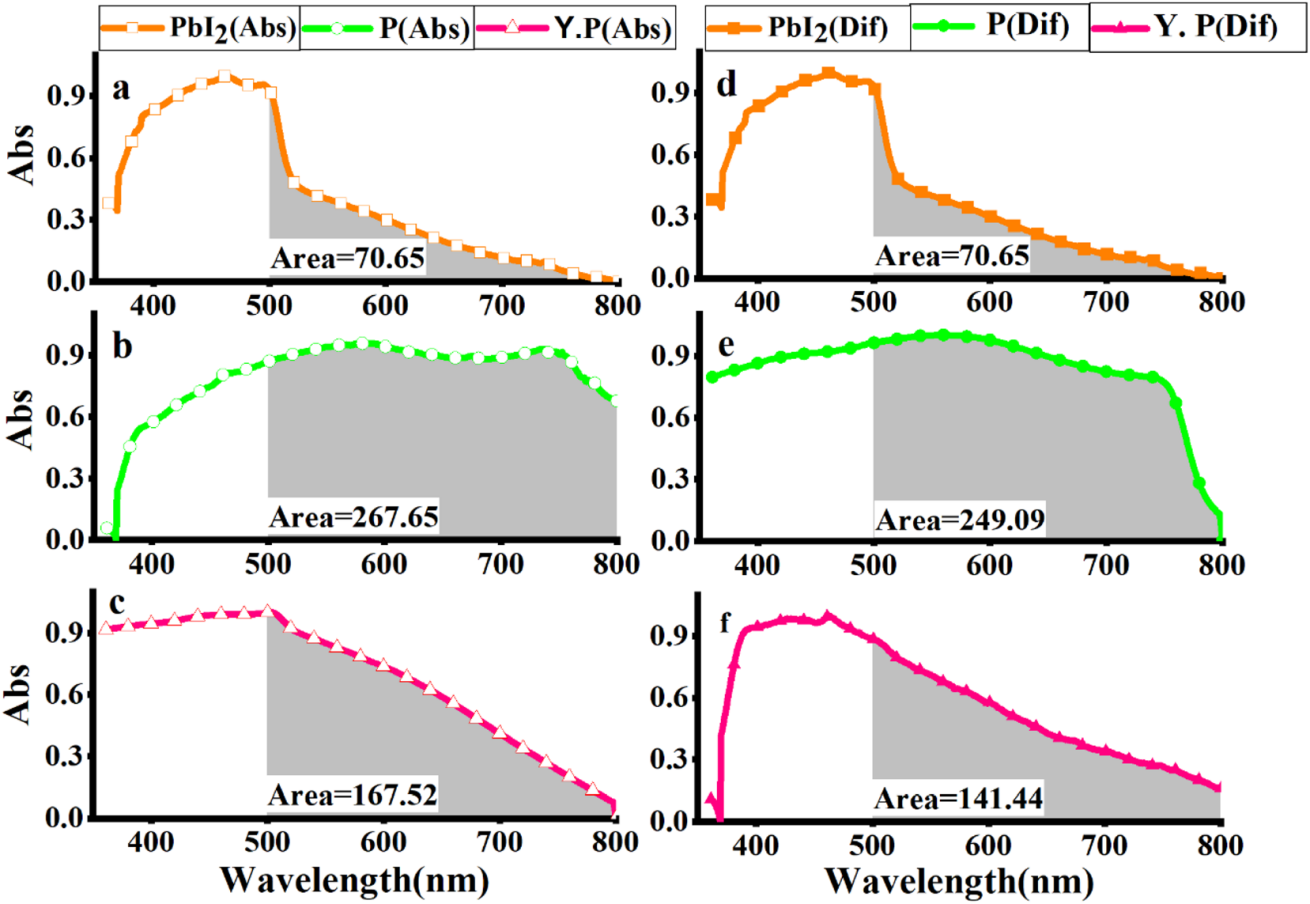
UV-vis absorption spectra of (a) PbI_2_, (b) perovskite film CH_3_NH_3_PbI_3_, (c) perovskite film CH_3_NH_3_PbI_3_ after two weeks, and UV-vis diffusion spectra of (d) PbI_2_, (e) perovskite film CH_3_NH_3_PbI_3_, and (f) perovskite film CH_3_NH_3_PbI_3_ after two weeks (Y. P.).

Since the light absorption range of perovskite as a light absorber in PSC is of particular importance, the absorption spectra of three compounds, PbI_2_, perovskite film CH_3_NH_3_PbI_3_ (P), and perovskite film CH_3_NH_3_PbI_3_, after two weeks were examined by absorption and diffusion reflectance spectroscopy. As shown in Fig. 4a and d, the absorption wavelengths of PbI_2_ extended from 380 to 800 nm, which experienced a sharp decline in the region 500–800 nm, while in the perovskite made with a molar ratio of 5, MAI (2), the intensity of this region increased dramatically, which is the main feature of perovskite films. The calculated sub-peak area of the highlighted range, calculated by Origin software, is about 70.71 for PbI_2_, while the sub-peak area of the perovskite significantly increased to 267.65, showing a 3.8-fold increase, as shown in Fig. 4(b). Then, when the perovskite film was maintained in the air for two weeks, the intensity of light absorption spectra decreased, and its area fell from 167.52 to 141.44, as shown in Fig. 4(c). In other words, when perovskite was converted to a yellow perovskite, the amount of light absorption decreased from 89.2% to 55.8%, which was also approved by results from diffusion reflectance spectroscopy, as shown in Fig. 4(d)–(f).

**Fig. 5:**
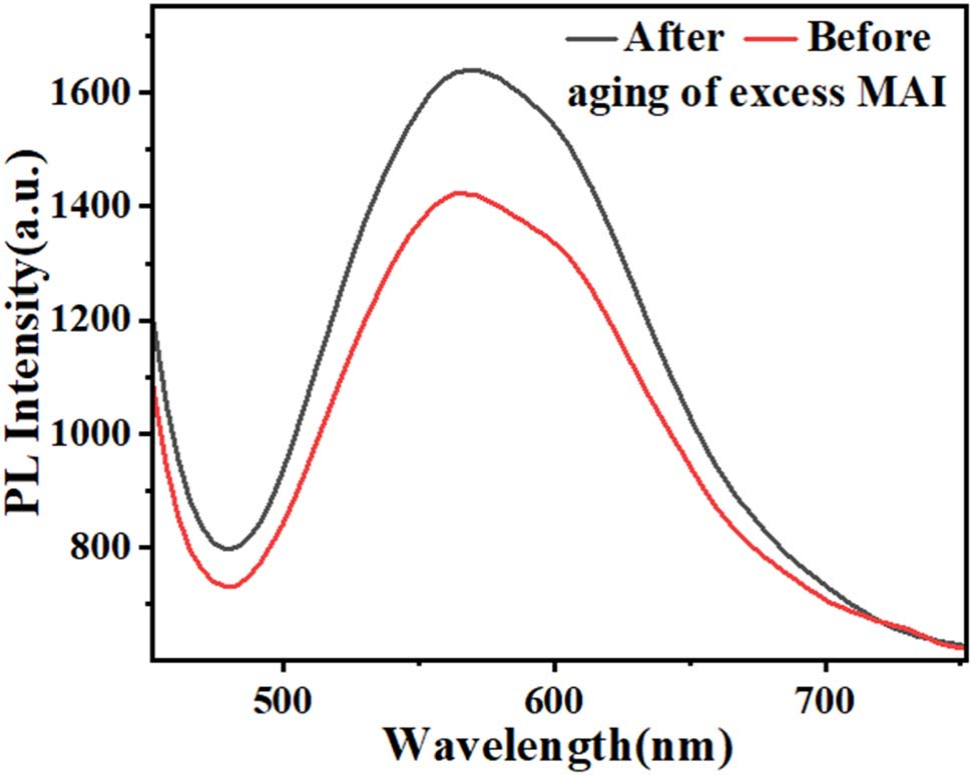
The optical properties (PL) of the films before and after aging of excess MAI.

Aiming to directly demonstrate the influence of the molar ratio of MAI on the excited states of perovskites samples, the photoluminescence spectroscopy of the films before and after aging of excess MAI was carried out, as shown in Fig. 5, confirming an increase in the emission intensity through the high molar ratio of MAI.

### 3.4. Stability measurements

**Fig. 6:**
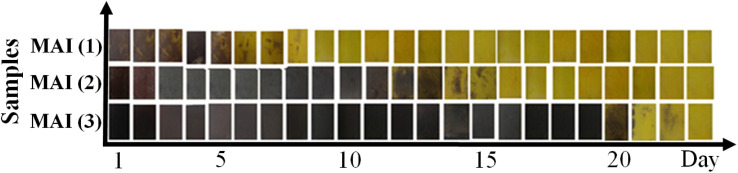
Stability test of perovskite film prepared with different concentrations of MAI, MAI (1), MAI (2), MAI (3).

Moreover, the stability test of perovskite film was studied based on different concentrations (1, 5, 25) M, MAI (1), MAI (2), and MAI (3) without any masking or encapsulation on the glass exposed to the air. As shown in Fig. 6, when the 1 : 1 molar ratio of MAI : PbI_2_ is used^42^ MAI (1), after about 5–6 days, the perovskite film begins to decompose and turns yellow. In comparison, the same perovskite film increases the stability of the film more than two times when the concentration of MAI increases from 1 to 5 M. This means that the prepared perovskite film, with a ratio of 1 : 5 of PbI_2_ : MAI, MAI (2), remains stable and without damage for about 13 days. Finally, with the increase in the MAI molar ratio to 25 M, MAI (3), the perovskite film remains stable, and this stability is fixed in the presence of air and humidity for up to 20 days. These observations indicated that the higher molar ratio of MAI by 25 M contributes significantly to the stability of the perovskite films.

**Fig. 7:**
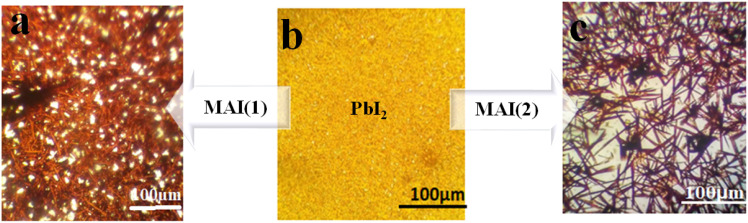
Schematic illustration of optical microscope images of perovskite thin films with different ratios of MAI (a) MAI (1), (b) PbI_2_ (c) MAI (2).

Moreover, photography images by optical microscopy are shown in Fig. 7. Glass cover with yellow PbI_2_ creates different crystal morphology patterns of MAI (1) and MAI (2). It is useful to note that after a comparison of about 100 samples, we proved that the image of a pure perovskite has a needle structure, as indicated in Fig. 7c, introducing a facile tool to demonstrate the provided perovskite by benchmark optical microscopy.

**Fig. 8:**
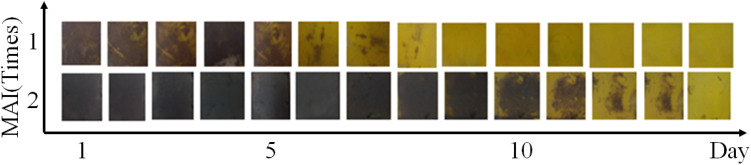
Stability test of perovskite film prepared with MAI (1 M) spin-coated once and twice.

As shown in Fig. 8, if MAI was spin-coated on the PbI_2_ twice, the stability of the perovskite film increased compared to once.

**Fig. 9:**
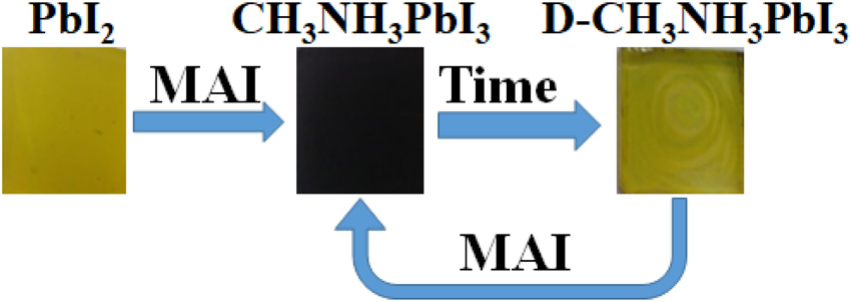
Schematic illustration of optical microscope image reversibility of perovskite destruction reaction.

**Fig. 10:**
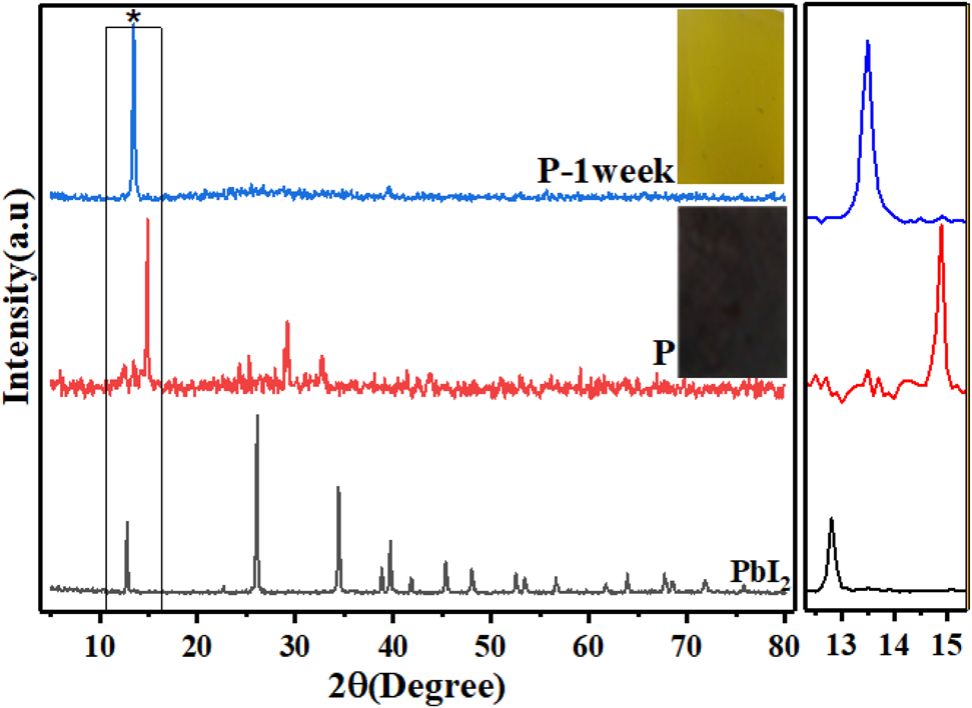
XRD pattern of PbI_2_, (P) perovskite, and (P-1 week) decomposed perovskite.

In particular, when perovskite film decomposes to yellow perovskite and PbI_2_, reversibly by adding excess MAI to the surface by spin-coating, the perovskite film is formed again. The evidence presented in Fig. 9 confirms that the excess MAI could efficiently influence generating perovskite continuously, which enhances the stability of perovskite film (Fig. 10).

**Fig. 11:**
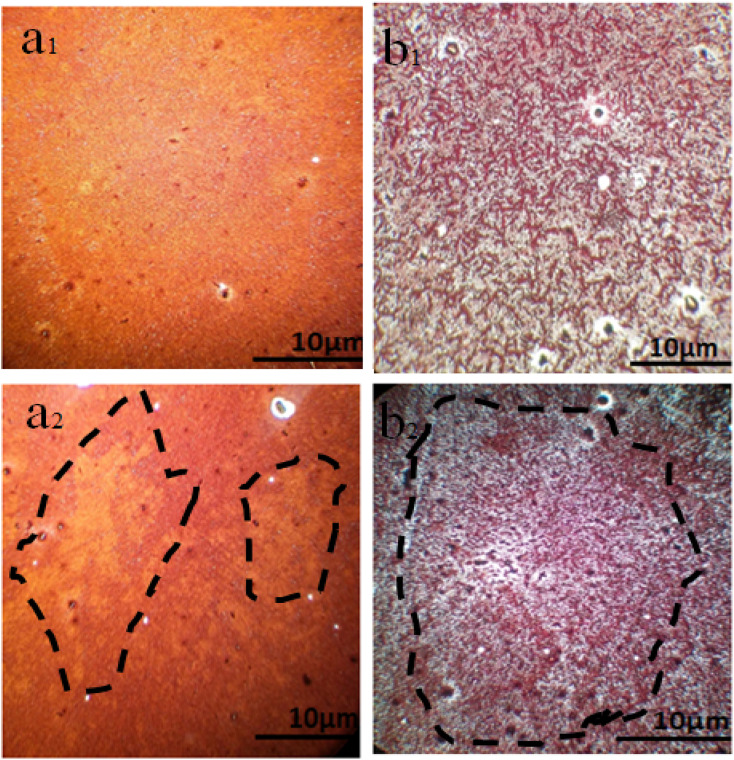
Optical microscope image of perovskite thin films with different ratios of (MAI), (a_1_) MAI (1) fresh, (a_2_) MAI (1) after 24 h, (b_1_) MAI (3) fresh, (b_2_) MAI (3) after 24 h.

To illustrate the changes in the perovskite film after 24 h, let us look at the images taken with the optical microscope, as shown in Fig. 11. If PbI_2_ film is covered by MAI (1 M) to perovskite, MAI (1), Fig. 11(a_1_), after 24 h, the morphology of films changes to that shown in Fig. 11(a_2_), appearing as yellow islands, which indicates the beginning of perovskite decomposition. While by using excess MAI to cover the perovskite MAI (25 M), MAI (3), after the same time, the density of perovskite grains was extended, converting from Fig. 11(b_1_) and (b_2_).

**Fig. 12:**
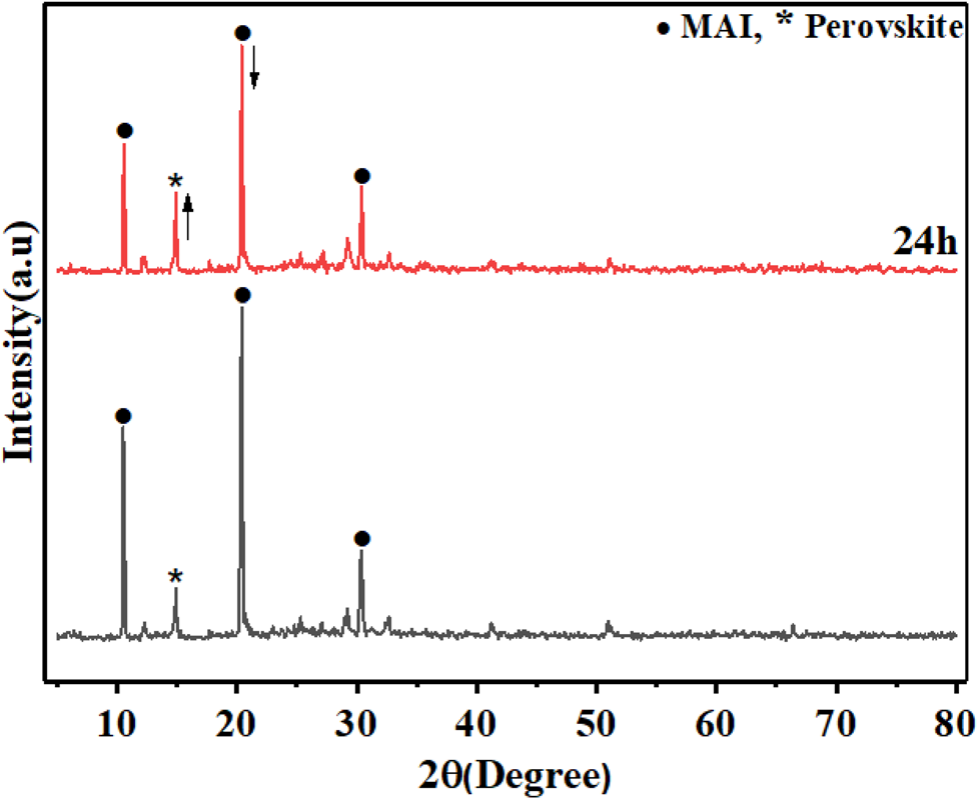
XRD pattern of MAI (3) fresh and 24 h later.

To study the effect of MAI concentration on the stability of perovskite film, a further survey of the perovskite prepared with MAI (25 M), MAI (3) by XRD measurements was done. Fig. 12 shows the XRD pattern of MAI (3) and 24 h later. Surprisingly, after 24 h, the peak height of the main perovskite surface 110 located at 14.9° increased, indicating the growth of perovskite grains, which means that perovskite crystallite did not decompose and increased the quality of the prepared perovskite layer. Moreover, the intensity of MAI's peak centered at 20.4° reduced after 24 h compared to that in the beginning, which indicated that the excess MAI significantly influenced the quality of crystallinity of perovskite.

**Fig. 13:**
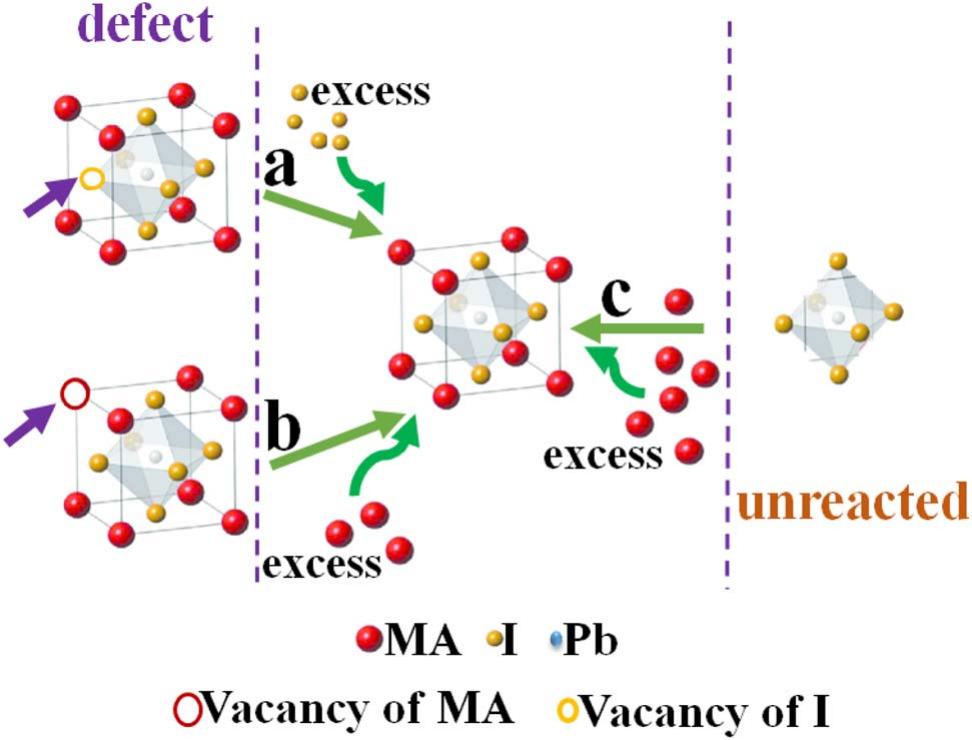
Schematic mechanism of (a and b) the effect of excess MAI on the compensation of perovskite crystal defects and (c) converting the remaining PbI_2_ to perovskite crystals.

Finally, the process of forming a perovskite film is not completely uniform and always some vacancies for cations and anions as well as PbI_2_ that have not entered the crystallization process of perovskite exist. As shown schematically in Fig. 13, the mechanism of the increasing amount of the density of perovskite crystals seems to be due to the presence of excess MAI to retrieve perovskite crystal defects (a and b) through reaction with the remaining PbI_2_ to re-crystallization of perovskite film (c).

## 4. Conclusion

In summary, we found that the high molar ratio of the MAI component compared to PbI_2_ can efficiently improve the stability and crystallinity of the perovskite structure. The proposed mechanism shows that methylammonium is charging the perovskite structure after the decomposition of perovskite to PbI_2_ over time, leading to perovskite stability, which is a disadvantage in the perovskite field. Indeed, excess MAI with a 25 : 1 ratio to PbI_2_ precursor gradually contributes to the restriction of perovskite film, which is confirmed by XRD and XPS analyses. In particular, it is found that high purity and crystallinity of perovskite could be obtained *via* the optimized molar ratio of 1 : 5 of PbI_2_ to MAI. This new evidence opens new ways of understanding perovskite decomposition and finding a simple way to address this huge challenge in PSC.

## Conflicts of interest

There are no conflicts to declare.

## Supplementary Material

RA-013-D3RA01304A-s001
