# Supplementary material for: Mechanistic insights into the key role of methylammonium iodide in the stability of perovskite materials
Source: RSC Adv. 2023 Jul 10;13(30):20408–16. doi: 10.1039/d3ra01304a (PMC10331566; doi:10.1039/d3ra01304a)
Supplement: RA-013-D3RA01304A-s001 [file RA-013-D3RA01304A-s001.pdf]

Electronic Supplementary Material (ESI) for RSC Advances.
This journal is © The Royal Society of Chemistry 2023

Supplementary Information

Mechanistic insights of the key role of methylammonium iodide in the stability of perovskite material

Negin Sabahi and Hashem Shahroosvand*

Table S1: History of studies related to MAI

		PbI_2/MAI	Additive	Condition	Ref.	Year
Methylammonium iodide	Precursor	1, 1	$H_2O_{(Trace)}$	$MAI_{Assay}(H_4P_2O_6)_{dipping}$	1, 2 3	2016-2017-2016
		1,1, 1	HI, CH_3I	$MAI_{loading\ time,\ co\text{-}evaporation}$	2, 4 5	2016-2015-2016
		1/3	DMSO	$MAI_{Vapor\text{-}based}$	6 7	2021-2020
		1		$MAI_{Purity,Vapor\text{-}Phase}$	8	2022
		1		$PbI_{2powder}$+MAI	9	2016
		1/4		Precursor $_{Temperature,\ one\ step}$	10	2021
		1/2, 1/3, 1/5		$MAI_{time\ of\ dipping}$	11	2018
		1		MAI-based $_{co\text{-}evaporation}$	12	2019
		1		MAI $_{aged}$	13	2021
		1		MAI $_{co\text{-}evaporation}$	14	2016
		-		Computational details	15	2019
		1		PbIx:MAAc:MAI	16	2017
		1		BAI:MAI:PbI_2:NH_4SCN	17	2017
		1		Synchrotron source	18	2022
		1		Thermal co-evaporation	2	2016
		1		Phase diagram $_{one\ step}$	19	2017
	Post-syn	10%	MAI_{excess}+ $CsPbI_2Br$		20	2020
		-10% to 10%		$MAI_{extra\text{-}stoichiometric}$	21	2019
		(10,30)mM		MAI $_{post\text{-}treatment}$	22 23	2019-2022
		Partial pressure		MSGG	24	2021

References:

1. C. Aranda, C. Cristobal, L. Shooshtari, C. Li, S. Huettner and A. Guerrero, *Sustainable Energy & Fuels*, 2017, **1**, 540.
2. N. Adhikari, A. Dubey, E. A. Gaml, B. Vaagensmith, K. M. Reza, S. A. A. Mabrouk, S. Gu, J. Zai, X. Qian and Q. Qiao, *Nanoscale*, 2016, **8**, 2693.
3. I. Levchuk, Y. Hou, M. Gruber, M. Brandl, P. Herre, X. Tang, F. Hoegl, M. Batentschuk, A. Osvet and R. Hock, *Advanced Materials Interfaces*, 2016, **3**, 1600593.
4. T. G. Kim, S. W. Seo, H. Kwon, J. Hahn and J. W. Kim, *Physical chemistry chemical physics*, 2015, **17**, 24342.
5. Y. Cheng, H.-W. Li, J. Zhang, Q.-D. Yang, T. Liu, Z. Guan, J. Qing, C.-S. Lee and S.-W. Tsang, *Journal of Materials Chemistry A*, 2016, **4**, 561.
6. F. Sahli, N. Miaz, N. Salsi, C. Bucher, A. Schafflutzel, Q. Guesnay, L. Duchêne, B. Niesen, C. Ballif and Q. Jeangros, *ACS Applied Energy Materials*, 2021, **4**, 4333.

7. S. H. Lee, S. Hong, S. An, T.-Y. Jeon and H. J. Kim, *Electronic Materials Letters*, 2020, **16**, 588-594.
8. M. Roß, M. B. Stutz and S. Albrecht, *Solar RRL*, 2022, **6**, 2200500.
9. M. B. Johansson, T. Edvinsson, S. Bitter, A. I. Eriksson, E. M. Johansson, M. Göthelid and G. Boschloo, *ECS Journal of Solid State Science and Technology*, 2016, **5**, P614.
10. S. Raj Mohan, M. Joshi, T. Dhami, S. Rai and R. Singh, *Journal of Materials Science: Materials in Electronics*, 2021, **32**, 2459.
11. D. A. A. Leal, B. Krishnan, S. Shaji and D. A. Avellaneda, *Materials Chemistry and Physics*, 2018, **215**, 137.
12. R. Kottokkaran, H. A. Gaonkar, H. A. Abbas, M. Noack and V. Dalal, *Journal of Materials Science: Materials in Electronics*, 2019, **30**, 5487-5494.
13. S. Mandati, E. Ramasamy, S. Mallick, T. Rao and G. Veerappan, *Materials Letters*, 2021, **299**, 130056.
14. Y.-Y. Huang, G. Gollavelli, Y.-H. Chao and C.-S. Hsu, *Journal of Materials Chemistry C*, 2016, **4**, 7595.
15. L. Zhang, B. Wu, S. Lin and J. Li, *Russian Journal of Physical Chemistry A*, 2019, **93**, 2694.
16. S. Venkatesan, M. Hasan, J. Kim, N. R. Rady, S. Sohal, E. Neier, Y. Yaoc and A. Zakhidova, *Journal of Materials Chemistry C*, 2017, **5**, 10114.
17. X. Zhang, G. Wu, S. Yang, W. Fu, Z. Zhang, C. Chen, W. Liu, J. Yan, W. Yang and H. Chen, *Small*, 2017, **13**, 1700611.
18. Z. Inamul Hasan, S. Joshi and K. Subbaya, *Journal of Materials Science: Materials in Electronics*, 2022, **33**, 16369.
19. A. Mishra, D. Hodges and R. Misra, *Materials Research Express*, 2017, **4**, 096201.
20. K. S. Kim, I. S. Jin, S. H. Park, S. J. Lim and J. W. Jung, *ACS applied materials & interfaces*, 2020, **12**, 36228.
21. K. Liao, J.-a. Yang, C. Li, T. Li and F. Hao, *ACS applied materials & interfaces*, 2019, **11**, 39882.
22. A. Kogo, T. Miyadera and M. Chikamatsu, *ACS applied materials & interfaces*, 2019, **11**, 38683.
23. R. P. Srivastava, J. Lee and D.-Y. Khang, *Surfaces and Interfaces*, 2022, **29**, 101703.
24. H. Fan, J.-H. Huang, L. Chen, Y. Zhang, Y. Wang, C. Gao, P. Wang, X. Zhou, K.-J. Jiang and Y. Song, *Journal of Materials Chemistry A*, 2021, **9**, 7625.
